# Impact of Genetic Background on Allele Selection in a Highly Mutable *Candida albicans* Gene, *PNG2*

**DOI:** 10.1371/journal.pone.0009614

**Published:** 2010-03-09

**Authors:** Ningxin Zhang, Richard D. Cannon, Barbara R. Holland, Mark L. Patchett, Jan Schmid

**Affiliations:** 1 Institute of Molecular BioSciences, Massey University, Palmerston North, New Zealand; 2 Department of Oral Sciences, University of Otago, Dunedin, New Zealand; 3 Allan Wilson Centre for Molecular Ecology and Evolution, Massey University, Palmerston North, New Zealand; University of Texas-Houston Medical School, United States of America

## Abstract

In many microbes rapid mutation of highly mutable contingency genes continually replenishes a pool of variant alleles from which the most suitable are selected, assisting in rapid adaptation and evasion of the immune response. In some contingency genes mutability is achieved through DNA repeats within the coding region. The fungal human pathogen *Candida albicans* has 2600 repeat-containing ORFs. For those investigated (*ALS* genes, *HYR1, HYR2, CEK1, RLM1*) many protein variants with differing amino acid repeat regions exist, as expected for contingency genes. However, specific alleles dominate in different clades, which is unexpected if allele variation is used for short-term adaptation. Generation of new alleles of repeat-containing *C. albicans* ORFs has never been observed directly. Here we present evidence for restrictions on the emergence of new alleles in a highly mutable *C. albicans* repeat-containing ORF, *PNG2*, encoding a putative secreted or cell surface glycoamidase. In laboratory cultures new *PNG2* alleles arose at a rate of 2.8×10^−5^ (confidence interval 3.3×10^−6^−9. 9×10^−5^) per cell per division, comparable to rates measured for contingency genes. Among 80 clinical isolates 17 alleles of different length and 23 allele combinations were distinguishable; sequence differences between repeat regions of identical size suggest the existence of 36 protein variants. Specific allele combinations predominated in different genetic backgrounds, as defined by DNA fingerprinting and multilocus sequence typing. Given the *PNG2* mutation rate, this is unexpected, unless in different genetic backgrounds selection favors different alleles. Specific alleles or allele combinations were not preferentially associated with *C. albicans* isolates from particular body sites or geographical regions. Our results suggest that the mutability of *PNG2* is not used for short-term adaptation or evasion of the immune system. Nevertheless the large number of alleles observed indicates that mutability of *PNG2* may assist *C. albicans* strains from different genetic backgrounds optimize their interaction with the host in the long term.

## Introduction

Repetitive DNA sequences mutate several orders of magnitude faster than non-repetitive sequences through addition and removal of repeat units by strand slippage and recombination [1]. In many microbes such highly mutable repeat sequences are placed either upstream of, or within, ORFs where mutations may alter transcription rates or the amino acid sequence of the encoded proteins [2]–[4]. For the genes affected, often referred to as ‘contingency genes’, a pool of variant alleles is constantly replenished by repeat sequence mutation. From this pool the most suitable at a given time are selected, allowing rapid adaptation to changing circumstances and evasion of the immune response [2]–[4].

The genome of the human pathogen *Candida albicans* contains approximately 2600 repeat-containing ORFs, three and ten times more, respectively, than those of the ascomycete yeasts *Saccharomyces cerevisiae* and *Schizosaccharomyces pombe* [5]. Comparative genomic analyses of *C. albicans* strains suggest that repeat-containing ORFs may be important *C. albicans* fitness determinants [6]. To date, only a few of these genes have been characterized, including *EAP1*, *PIR1 CEK1*, *HYR1*, *HYR2, HWP1, RLM1* and the *ALS* (agglutinin like sequence) family of adhesins [7]–[16]. Allele variation has been assessed in *CEK1*, *HYR1*, *HYR2, RLM1* and the *ALS* family, and high allelic diversity, caused by variations in repeat regions, was observed in all of these ORFs [10]–[16]. The alleles differ in the number of highly conserved repeats and this translates into proteins differing in amino acid repeat numbers [10], [12]–[16]. An effect of changes in repeat numbers on the adhesive function of the protein has been demonstrated experimentally for Als5p [17]. Functions of the repeat regions in Hwp1p in adhesion [9], in Pir1p in localizing the protein [8], and in Eap1p in positioning binding sites of the encoded protein [7] have also been demonstrated.

To our knowledge the rate at which new alleles arise in repeat-containing *C. albicans* ORFs has never been determined. Hoyer and coworkers [18] and Zhao and coworkers [13] analyzed several *ALS* genes in serially passaged cultures but could not detect any new *ALS* alleles. For the related *S. cerevisiae FLO1* gene, alterations in its repeat region generate new alleles with a frequency of approximately 10^−5^ new alleles per cell per division [19], at the lower end of rates of changes in contingency loci [2], [20].

For all repeat-containing ORFs for which clade-specificity of alleles has been investigated (*ALS* genes, *HYR1, HYR2* and *CEK1*), alleles with particular repeat regions predominate in the different *C. albicans* clades in collections of isolates obtained from a variety of geographical regions, body sites and types of candidiasis [10], [12]–[15]. This is unexpected if these genes mediate rapid adaptation to changing circumstances or evasion of host defenses. Clades diverged more than 3 million years ago [21] whereas contingency genes can change so quickly that different alleles can predominate at different times during infection of a single host [20], [22]. Thus selection of alleles beneficial in specific host niches or selection of novel alleles in response to host defenses should have largely prevented any clade specificity. Clades are generally not confined in distribution to specific geographical regions, body sites or types of candidiasis [23], [24]; therefore association of particular alleles with specific geographical regions as a result of genetic drift or with specific host niches should also not generate clade specificity as a secondary consequence.

To better understand their biological role, we have begun to investigate additional repeat-containing ORFs in *C. albicans*. In a recent survey of DNA polymorphisms largely specific to a general-purpose genotype (GPG; equivalent to clade 1) of *C. albicans* strains, causing disease 10–100 times more often than other genotypes, we identified a GPG-specific polymorphism, caused by a GPG-specific number of repeats within the *PNG2* ORF [6].

*PNG2* encodes a putative peptide:*N*-glycanase (EC 3.5.1.52) [6], a class of enzymes that removes N-linked glycans from glycoproteins [25]. It is most likely secreted or located at the cell surface, as judged from sequence homologies with two experimentally verified peptide:*N*-glycanases [25], [26]. Its expression is upregulated in response to treatment with caspofungin, ciclopirox olamine or ketoconazole [27], [28], downregulated in response to lowered pH [29] and its transcript is detectable by reverse transcriptase PCR in oral candidiasis [6]. We describe here our characterization of the variability of the *PNG2* ORF repeat region and conclusions, based on these results, regarding the role of this variability in adaptation.

## Results

### Seventeen Different-Sized *PNG2* Repeat Regions Forming 23 Different Repeat Region Combinations in 80 Clinical *C. albicans* Isolates

**Figure 1:**
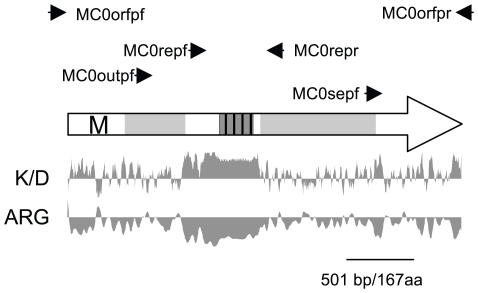
*PNG2* ORF and its predicted protein product, based on the published SC5314 genome sequence. The repeat region is shown grey and striped, regions with homology to glycoamidases are marked light grey and a predicted transmembrane helix (FCQILSLFGVLYLVNFLYI; amino acid residues 69-87) is marked with the letter M. Positions of primers used in this study are shown as arrows. KD: Kyte/Doolittle hydrophilicity plot; ARG: Argos Helix plot of the protein.

**Figure 2:**
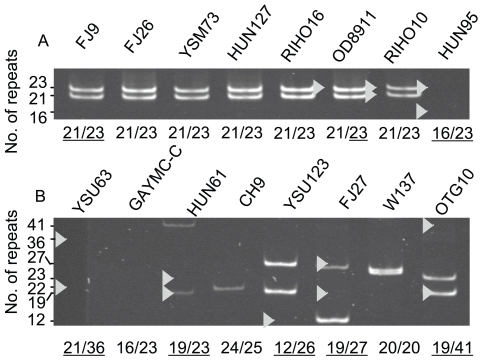
Examples of acrylamide gels used for assessment of repeat region sizes in *C. albicans* isolates. PCR products were amplified using primers MC0repf and MC0repr from genomic DNA of GPG strains (A) and other strains (B), and separated on acrylamide gels. Repeat numbers predicted from amplicon size are shown below the gels. Repeat numbers verified by sequencing are underlined. Sequence-verified PCR products, marked by white triangles, were also used as molecular weight markers.

The *PNG2* ORF first came to our attention because differences in the copy number of 12 bp repeats (encoding mainly PPHE and PPHH; Fig.1) created an amplified fragment length polymorphism (AFLP) largely specific to GPG clinical isolates [6]. We extended this analysis by PCR amplification of the repeat-containing region in 80 clinical isolates (Fig. 2, Table 1) to determine the number of different-sized alleles. We found 17 different-sized repeat regions containing between 12 and 41 repeat units. *C. albicans* is diploid and repeat regions combined to form 23 different pairwise allele combinations. We sequenced 31 repeat regions and confirmed that alterations in the number of repeat units were responsible for alterations in the size of the PCR product in every case, and that the PCR-based estimates of the number of repeats were correct in every case.

**Table 1:** *C. albicans* isolates and their *PNG2* repeat region size combinations.

Isolate^a^	Group^b^	Country of isolation	Isolation site^c^	No. of repeats^d^
FJ26	GPGA1	Fiji	s/w	21,23
FJ9	GPGA1	Fiji	r/o	21,23
FJ23	GPGA1	Fiji	u	21,23
YASU751	GPGA1	Malaysia	u	21,23
AU1	GPGA1	New Zealand	r/o	21,23
CH14	GPGA1	New Zealand	s/w	21,23
VAR1.1VAG	GPGA1	USA	v	21,23
VAR1.3VAG	GPGA1	USA	v	21,21
HUN122	(GPGA1)	Great Britain	s	24,25
CLB42	GPGA2	Colombia	v	20,23
CLB56	GPGA2	Colombia	s/w	21,23
CLB53	GPGA2	Colombia	s/w	21,23
FJ10	GPGA2	Fiji	r/o	21,23
OD8807	GPGA2	Great Britain	r/o	21,21
OD8911	GPGA2	Great Britain	r/o	21,23
HUN127	GPGA2	Great Britain	s	21,23
HUN95	GPGA2	Great Britain	s	16,23
OD8916	GPGA2	Great Britain	r/o	21,23
HUN93	GPGA2	Great Britain	s	21,21
HUN96	GPGA2	Great Britain	s	21,21
OD8826	GPGA2	Great Britain	r/o	21,23
YASU568	GPGA2	Malaysia	u	21,23
YASU649	GPGA2	Malaysia	u	21,23
YASM73	GPGA2	Malaysia	r/o	21,23
AU19	GPGA2	New Zealand	u	21,23
AU90	GPGA2	New Zealand	s/w	21,23
CH35	GPGA2	New Zealand	u	21,23
W132	GPGA2	New Zealand	r/o	21,23
W26	GPGA2	New Zealand	a	21,23
W43	GPGA2	New Zealand	r/o	21,23
CH42	GPGA2	New Zealand	s/w	21,23
CHOB5	GPGA2	New Zealand	s/w	21,23
JAM-2C	GPGA2	USA	a	23,23
RIHO10	GPGA2	USA	s	21,23
COUR-C	GPGA2	USA	r/o	21,23
RIHO13	GPGA2	USA	s	21,23
VAR1.4VAG	GPGA2	USA	v	21,23
RIHO16	GPGA2	USA	s	21,23
RIHO9	GPGA2	USA	s	21,23
VAR1.10VAG	GPGA2	USA	v	21,23
VAR1.8VAG	GPGA2	USA	v	21,23
HUN123	B	Great Britain	s	23,23
HUN61	B	Great Britain	r/o	19,23
AU2	B	New Zealand	r/o	21,21
W137	B	New Zealand	r/o	20,20
CH9	B	New Zealand	v	24,25
OTG18	B	New Zealand	r/o	23,23
VAR1.7VUL	B	USA	v	21,23
VAR1.5VAG	B	USA	v	23,23
RIHO30	B	USA	s	16,20
CLB49	C	Colombia	r/o	21,36
CLB44	C	Colombia	s/w	19,27
CLB45	C	Colombia	s/w	22,27
FJ12	C	Fiji	r/o	19,41
FJ27	C	Fiji	c	19,27
HUN68	C	Great Britain	r/o	22,23
HUN64	C	Great Britain	s/w	19,41
OD8824	C	Great Britain	r/o	21,26
HUN91	C	Great Britain	s	18,20
HUN66	C	Great Britain	s/w	21,23
YASU123	C	Malaysia	u	12,26
YASU363	C	Malaysia	u	21,36
YASU63	C	Malaysia	u	21,36
YASM1	C	Malaysia	s/w	22,26
YASU709	C	Malaysia	u	23,23
YASM42	C	Malaysia	r/o	21,36
CH3	C	New Zealand	u	22,23
W142	C	New Zealand	r/o	16,16
AU36	C	New Zealand	c	22,23
OTG10	C	New Zealand	a	19,41
W53	C	New Zealand	r/o	19,41
AU134	C	New Zealand	r/o	19,41
W17	C	New Zealand	r/o	17,27
W55	C	New Zealand	a	19,41
CH20	C	New Zealand	v	16,41
OTG1	C	New Zealand	r/o	23,23
OTG4	C	New Zealand	v	19,37
GAYMC-C	C	USA	r/o	15,23
SW-17C	C	USA	s	19,27
HUN92	(C)	Great Britain	s	21,23
SC5314	GPG^e^	laboratory strain		21,23

a See [23] for more detail on isolates.

b A, B and C are the three major jackknifing-supported branches within the species, with limited support for a further subdivision of the general-purpose genotype A into two groups A1 and A2, based on Ca3 fingerprints [23]; for strains in brackets other markers are in conflict with the Ca3-based classification [6].

c Strains were isolated from: anal sites (a), catheter (c), respiratory and oral sites (r/o), skin and wounds (s/w), sterile sites (s), urine (u), vagina/vulva (v).

d Number of repeat units in both alleles based on length of PCR products (and for some strains also by sequencing; see Fig. 5); where only one product was detected it was assumed that the isolate possessed two identical-sized repeat units.

e Based on [30].

In several *ALS* genes the differences in repeat numbers between the two alleles present in a given isolate differ significantly from those expected if all observed alleles were randomly paired with each other [13], [15]. This was not the case for *PNG2*. Differences in repeat numbers between the two copies of the gene in a given strain covered a wide range (0–25 repeats), and the average difference in the number of repeat units (5 units) did not differ significantly from the differences observed in 1000 simulations in which alleles were randomly combined into 80 pairs.

### Different Alleles and Allele Combinations Dominate in Different Genetic Backgrounds Regardless of the Body Site or Geographical Region from Which Isolates Were Obtained

**Figure 3:**
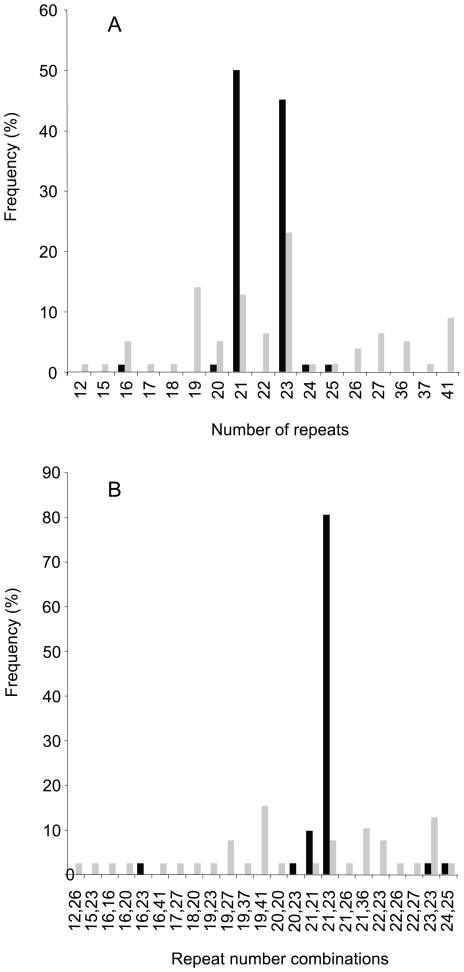
Frequencies of repeat regions of different lengths and frequencies of repeat region combinations. Frequencies (B) among GPG clinical isolates are indicated by black bars, frequencies among other clinical isolates by grey bars. Lengths (A) are expressed as numbers of 4 amino acid repeat units. One borderline GPG strain and one borderline non-GPG strain (see Table 1) were included in the analysis.

Forty-one of our clinical isolates belonged to the GPG cluster and 39 belonged to a variety of other genotypes ([23], Table 1). Two repeat region lengths were overrepresented in GPG isolates (Fig. 3A). Fifty percent of all GPG alleles had a 21 unit repeat region and 45% had a 23 unit repeat region. Only 13% and 23% of alleles from other isolates had 21 and 23 repeat unit lengths, respectively (*z* test, *P*<0.05 with Bonferroni correction and assuming that isolates with only one repeat unit size detectable by PCR carried two units of identical size). Even though they were less frequent than in GPG strains, both the 21 and the 23 repeat lengths were more common than most other repeat lengths among 39 non-GPG isolates analyzed. Non-GPG strains represent a spectrum of highly diverse *C. albicans* genotypes [21], [23], [24] but can be subdivided into two major jackknifing-supported evolutionary branches, B and C [23]. The 23 unit repeat was significantly more frequent in branch B than in branch C (8/18 alleles versus 10/60 alleles respectively; *z* test, *P*<0.05, Table 1).

A more striking difference between GPG strains and other strains occurred in terms of allele combinations (Fig. 3b). The repeat combination 21 plus 23 repeats was ten times more frequent in GPG than in non-GPG clinical isolates (33 out of 41 (80%) versus 3 out of 39 (8%); *z* test, *P*<0.001). The next most common allele combination in GPG strains was 21 plus 21 repeats (10% of isolates), very similar to the 21 plus 23 combination. There was no clearly predominant allele combination among non-GPG strains.

We found no evidence that geographical region or body site of isolation affected the frequencies of alleles or allele combinations. Among GPG strains the 21 plus 23 repeat combination predominated in all geographical regions and in all body sites from which GPG isolates had been obtained (Table 1). There was no indication that GPG isolates with other allele combinations were preferentially obtained from a specific geographical region or body site (Table 1). Among non-GPG strains, all allele combinations that occurred more than once were found in isolates from different geographical regions and different body sites. There was no indication that any allele combination was overrepresented in a specific region or among non-GPG isolates from a particular body site (Table 1).

We did not include laboratory strain SC5314 in these analyses. Unlike the clinical isolates we used, SC5314 has been propagated in the laboratory for many years under selective pressures likely to be different from those in the human host, and its repeat region may have changed as a result. Indeed, while our stock of the strain had the allele combination 21 plus 23 repeats, predominant in GPG strains (SC5314 is a GPG strain [30]), the published SC5314 genome sequence [31] has two alleles with 21 repeats.

### In Laboratory Cultures, the *PNG2* Repeat Region Generated New Alleles at a Frequency of 2.8×10^−5^ per Cell Division

**Figure 4:**
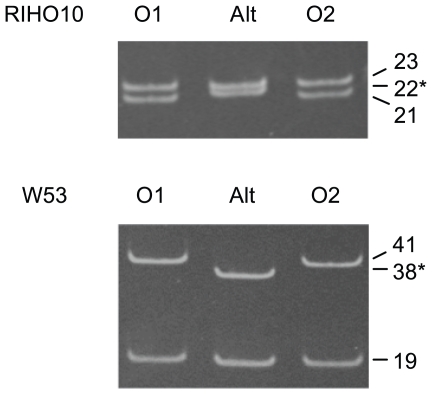
Alterations of repeat regions after 300 generations of serial transfer of strains RIHO10 and W53. PCR products amplified from genomic DNA using primers MC0repf and MC0repr were resolved on acrylamide gels. For each strain the middle lane shows PCR products from the clone with an altered allele (ALT), flanked by two lanes of PCR products from clones which retained alleles of the original lengths. Figures on the right show the numbers of repeats, with altered allele marked by an asterisk. Numbers of repeats were verified by sequencing (see Fig. 5).

**Figure 5:**
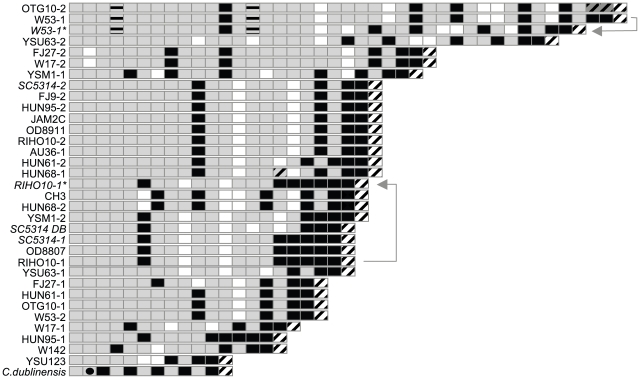
Graphic representation of the amino acid sequences of PNG2 repeat regions. The *PNG2* repeat regions of 33 *C. albicans* strains were sequenced and the predicted amino acid sequences, plus the repeat region from the *C. dubliniensis* ortholog (gi241951534), were compared. Grey boxes represent the repeat PPHE, black boxes PPHH, and unfilled boxes PPHK. Grey boxes with diagonal black stripes represent PPHP, grey boxes with horizontal black stripes represent SPHE, white boxes with diagonal grey stripes PPPE, white boxes with diagonal black stripes PPHD and checkered boxes PSHH. The *C. dubliniensis*-specific repeat PGDK is represented by a grey box with a black dot. Where two alleles of the same strain were sequenced, the name of the strain is followed by a dash followed by the allele number (1 or 2, with 1 being the allele with the shorter repeat region). Names of laboratory strains are italicized, including the derivatives of the serially transferred W53 and RIHO10 with altered alleles; the altered alleles are marked by asterisks and linked by an arrow to the allele in the original strain from which they are most likely derived. For laboratory strain SC5314 two alleles sequenced by us, and the sequence determined as part of the *C. albicans* genome sequencing project, “DB” are shown (both alleles in the genome database are identical in terms of amino acid repeats).

Repeat-containing ORFs in *C. albicans* should generate new alleles at rates comparable to those measured in other organisms, but this has so far not been observed. To measure the rate at which new *PNG2* alleles arise, we transferred four strains (two GPG strains, HUN93 and RIHO10 and two non-GPG strains, W53 and W142) serially for 300 generations and screened 60 colonies per strain by colony PCR. Two of the 240 colonies had one altered allele each. In one RIHO10 progeny colony, one allele had gained one unit, and in one W53 progeny colony, one allele had been shortened by 3 repeat units (Figs 4, 5). Thus new *PNG2* alleles had been generated by insertions and deletions of repeat units with a frequency of 2 in 72,000 cell divisions (2.8×10^−5^; confidence interval 3.3×10^−6^−9. 9×10^−5^) comparable to that observed by Versterpen and coworkers for the repeat-containing *FLO1* gene in *S. cerevisiae* [19].

### Evidence That the Repeat Region Has a Biological Function

The function of the *PNG2* ORF is unknown. We have proposed the name *PNG2* based on a bioinformatics analysis suggesting that it encodes a non-cytoplasmic (i.e. secreted or cell surface-located) peptide:*N*-glycanase (peptide-*N* ^4^-(*N*-acetyl-b-D-glucosaminyl)asparagine amidase, EC 3.5.1.52). N- and C-terminal regions of Png2p flanking the repeat region have significant amino acid sequence similarity to two eukaryotic secreted peptide:*N*-glycanases with experimentally verified enzyme activity, PNGase At from *Aspergillus tubingensis* [25] (E = 1×10^−21^ for the C-terminal region, and 4×10^−18^ for the N-terminal region), and PNGase A from *Prunus dulcis* (almonds) [26] (GI:56405352; E = 4×10^−20^ for the C-terminal region, and 3×10^−14^ for the N-terminal region), but not to the *S. cerevisiae PNG1* intracellular peptide:*N*-glycanase and its *C. albicans* ortholog ORF 19.26. A predicted transmembrane helix (Fig. 1) could serve as both membrane anchor and internal signal sequence [32], [33], and the bulk of *PNG2* orthologs from 52 fungi are predicted to be non-cytoplasmic (either secreted or anchored in the cell membrane). Like its well-characterized orthologs, *C. albicans* Png2p has multiple potential sites for *N*-linked glycosylation, which can only occur during translocation into the endoplasmic reticulum [34] as part of the secretory pathway.

The repeat region of Png2p is not present in any non-*Candida* orthologs. It interrupts the conserved regions of Png2p but such an interruption would not *per se* preclude the *Candida* protein from functioning as a non-cytoplasmic peptide:*N*-glycanase. During posttranslational processing of the *P. dulcis* peptide:*N*-glycanase, the two conserved regions are cleaved at a position corresponding to that of the repeat region in *C. albicans* Png2p to form the active heterodimeric enzyme [26].

**Figure 6:**
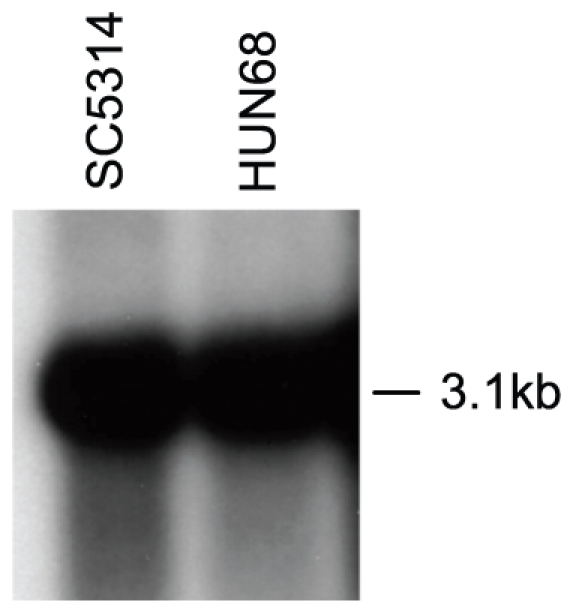
Verification of *PNG2* expression by Northern hybridization. Messenger RNA (5 µg per lane) prepared from exponential phase cultures of SC5314 and HUN68 grown in YPD was hybridized under high-stringency conditions with a probe corresponding to the repeat region.

Evidence that the *PNG2* ORF is transcribed comes from transcriptome microarray studies and reverse transcriptase PCR [6], [27]–[29], and we confirmed this by Northern hybridization (Fig. 6). PolyA-RNA hybridized with a repeat region probe revealed a 3.1 kb band consistent with the 2.95 kb size of the *PNG2* ORF. Png2p, however, has to our knowledge never been detected in proteomic investigations (based on the literature and on searches of mass spectrometry data by C.A. Munro, J. L. Lopez-Ribot, D. Thomas and C. Gil). Our own attempts to demonstrate the presence of the protein and its location by translational fusion with GFP [35] failed. No transformants were obtained in several attempts to replace one copy of the gene with an ORF encoding the fusion protein, even though heterozygous deletion mutants are viable [36] (data not shown; the construct was confirmed by sequencing).

We therefore sought evidence for a biological function of the protein and its repeat region through comparative sequence analysis. We sequenced five alleles of the entire ORF (both copies of *PNG2* in strains W53 and HUN68 plus one allele of our stock of SC5314) and found that outside of the repeat regions, nucleotide sequences were >99% identical, as is the case in *C. albicans* housekeeping genes [37]. The average ratio of non-synonymous to synonymous mutations (dN/dS) distinguishing pairs of sequences outside the repeat-containing region was 0.37, which is comparable to that of *C. albicans* housekeeping genes [38]. In 9 out of 10 comparisons of pairs of sequences the ratio was less than 1.0. A ratio of <1 is an indicator of purifying selection i.e. functionality of the encoded protein, whereas a ratio of 1 would indicate no selection [39]. We confirmed that the observed dN/dS ratio was significantly (*P*<0.01) below 1 by a likelihood ratio test comparing the fit between a model using the observed dN/dS ratio and the fit between the data and a model in which dN/dS was set to 1.0 [40].

The dN/dS ratio of repeat regions of different lengths cannot be easily assessed. Repeat regions would have to be trimmed to identical size prior to analysis. There are often many equally probable alignments of two repeat regions and thus there are no obvious criteria for deciding which unit should be removed in order to arrive at the correct dN/dS ratio. Calculating the dN/dS ratio for several hundred sequences encoding individual four amino acid repeats in all repeat regions is also not feasible. However, other types of sequence analysis provided several lines of evidence for the functionality of *PNG2* repeat regions at the protein level. Firstly, non-synonymous mutations were not randomly distributed in the repeats. Among all 28 sequences from the clinical isolates shown in Fig. 5, the frequency of amino acid replacements among the first three amino acids of the 4-amino acid repeats was 0.4% (7/1994 amino acids did not conform with the prevailing PPH motif). In contrast, it was 34% in the last amino acid (225/648 residues did not correspond to the most common amino acid E; *z* test, *P*<0.001); this asymmetry is not expected in the absence of selection since it implies significantly stronger purifying selection of first three amino acids, compared to the last. Also, there was an uneven distribution of the different types of repeat units through the repeat region, which would not be expected unless maintained by purifying selection: For instance, 66% of the five units preceding the terminal PPHD in Fig. 5 encode PPHH whereas only 8% of all repeats upstream of this region in Fig. 5 encode PPHH (*z* test, *P*<0.001); likewise PPHK units constitute 29% of the next five units upstream, compared to only 5% of all other units, a significant concentration in this region (*z* test, *P*<0.001).

That the repeat region is of functional significance at the protein level was also suggested by the presence of a very similar repeat region in the Pngp2 ortholog of *C. albicans*' sister species *Candida dubliniensis* (accession number **gi241951534**; 80% sequence identity, 90% sequence identity outside of the Pngp2 repeat region). As in *C. albicans,* (i) most units (11/12) start with PPH, (ii) the most common repeats are PPHE and PPHH and (iii) the last unit is PPHD (Fig.5). Such conservation would not be expected unless the repeat region has functional significance at the protein level. Likewise, given the high rate at which new alleles are generated in laboratory cultures, the overrepresentation of a specific allele combination in GPG strains is also not easily explained in the absence of selection.

## Discussion

Amino acid sequence similarity to the two known secreted peptide:*N*-glycanases (*P. dulcis* PNGase A, *Aspergillus tubingensis* PNGase At) suggests that Png2p is a peptide:*N*-glycanase. A multiple sequence alignment of PNGase A, PNGase At and *C. albicans* Png2p clearly identifies two conserved regions flanking the repeat region in Png2p. While the repeat region is absent in the two known secreted enzymes and their non-*Candida* orthologs, nonrandom distribution of amino acid replacements in the repeat region, the existence of clade-specific alleles and the high degree of its conservation between *C. albicans* and its sister species *C. dubliniensis* provide evidence (albeit indirectly) that the repeat region of the protein is of functional significance.

A possible function of the repeat regions is suggested by the heterodimeric nature of *P. dulcis* PNGase A. The position of the repeat region in *C. albicans* Png2p corresponds to the position at which the *P. dulcis* proenzyme is cleaved to generate the active heterodimer [26]. Conceivably the *P. dulcis* enzyme must be cleaved so that the resulting two subunits can be correctly positioned to form the catalytically competent enzyme. If so, the repeat units may mediate this positioning in the *C. albicans* Png2p. Altering the length of the repeat region could then alter the interactions between the conserved N- and C-terminal domains, and so modulate the enzyme's catalytic properties, in particular its substrate specificity. If Png2p is secreted, or located on the cell surface, as our bioinformatic analysis indicates, adhesion could be an additional function of the protein, mediated by a highly hydrophilic region, including the proline-rich repeats (with alterations of the repeat regions modulating adhesion). Proline-rich repeat motifs in fungal and bacterial proteins can mediate adhesion to host cells [41], [42], most notably the *C. albicans* Hwp1 protein [9]. As other *C. albicans* adhesins are known to have enzymatic activity [43], [44], Png2p could be an adhesin that removes glycans to expose host adherence receptors.

While we can only speculate on the exact function of Png2p and the functional significance of the repeat region, the fact that *PNG2* is transcribed and that the protein is under purifying selection are evidence that Png2p contributes to *C. albicans*' fitness, i.e. that it is a functional repeat-containing protein. As such it is a suitable object for expanding our knowledge on the role of allelic variation in repeat-containing *C. albicans* proteins.

*PNG2* behaves like a contingency gene in terms of the variety of alleles among 80 clinical isolates surveyed. Taking only repeat unit length into account we found 17 alleles. Sequencing increased this number to 24, due to differences in the arrangements of amino acid repeat units between regions of the same size in different strains (Fig. 5). As sequencing seven repeat region sizes from different strains revealed 14 different amino acid sequences (Fig. 5), we predict there could be approximately 36 variants of the protein among the 80 clinical isolates we surveyed (Fig. 5). All of the differences between the sequenced alleles were caused by addition, removal and rearrangement of repeat units as expected if strand slippage and recombination are the predominant mechanisms that generate new alleles [1]. In contrast, the non-repetitive parts of the *PNG2* ORF sequences from different strains were >99% identical at the nucleic acid level, comparable to the level of sequence diversity in housekeeping genes [37], with most of the point mutations being synonymous.

New alleles were generated in laboratory cultures, also by addition and subtraction of repeat units, at an apparent frequency of 2.8×10^−5^ (confidence interval 3.3×10^−6^−9. 9×10^-5^) per cell per division, or 1.4×10^−5^ per locus per cell per division. This is comparable to that measured in *S. cerevisiae* for the repeat- containing *FLO1* [4], and at the lower end of the range of frequencies measured for contingency genes in other organisms [2], [20]. There are several reasons to assume that the true rate at which new *PNG2* alleles are generated exceeds the rate we observed. Firstly, our assay could not detect new alleles in which rearrangements of repeat units occurred without affecting repeat region length. Secondly, some new alleles will have been eliminated by genetic drift during serial propagation of the cultures. Lastly, if a change in *PNG2* alleles impacts on fitness under laboratory culture conditions, some newly generated alleles might have been reduced in frequency by competition with cells that retained the original allele. Nevertheless the rate we measured is 150 times the expected frequency for generation of new alleles by a single point mutation (8.8×10^−8^ new alleles per locus per generation, based on the point mutation rate in *S. cerevisiae* of 3.3×10^−10^ mutations per bp [45], and an average size of 267 bp of the repeat region in the four strains used in the transfer experiment). The results regarding the frequency of new *PNG2* allele generation are not necessarily at odds with those of Hoyer and colleagues who could not detect new alleles in the repeat-containing *C. albicans ALS* genes after 530 and 3000 generations [13], [18]. These authors analyzed culture aliquots rather than multiple single colonies after serial passage of the original strain; extrapolating from the rate at which new *PNG2* alleles arise, it can be estimated that even after 3000 generations 70–80% of the alleles in their cultures would have still been identical to those of the original strain (see *Materials* and *Methods* on details of calculation). A multitude of additional newly generated alleles, each present at low frequency, would have been difficult to detect.

The observed frequency of new allele generation is in apparent conflict with the association of specific *PNG2* alleles or allele combinations, regardless of body site and geographical origin of isolates, with specific genetic backgrounds. For instance, based on our laboratory experiments the 21 plus 23 unit repeat combination present in 80% of GPG strains should be reduced in frequency to 8% after 100,000 generations (the calculation is based on the very conservative assumption that only the 23 observed allele length combinations are possible). Using the rate of increase in cell numbers following inoculation of a rat model of commensal colonization as an indicator of doubling times in the host (1.4 doublings per day; R. D. Cannon, unpublished observations) 100,000 generations are equivalent to 190 years; based on the confidence limits of our laboratory measurements of rates of new allele generation, the time required to reduce the frequency to 8% could be between 50 and 1600 years. Thus only a very recent genetic drift event or strong selection in the GPG genetic background in the host would explain the high frequency of the 21 plus 23 combination in GPG strains. Genetic drift seems an unlikely explanation, firstly because the high number of alleles makes accidental predominance of one combination very unlikely, and secondly because under a genetic drift scenario we would expect the frequency of the 21 plus 23 combination to be less uniform between different geographical regions. Thus a strong impact of the GPG genetic background on the selection of *PNG2* allele combinations seems the most likely explanation for our results. Whether other genetic backgrounds in *C. albicans* also select in favor of specific allele combinations is more difficult to determine, because GPG strains are the only group of highly related isolates within the species that can be confidently defined with phylogenetic methods [23], [24]. However, we found some indications this may be the case because there was a significant difference in the frequency of the 23 unit allele between the two major branches of non-GPG strains. Likewise MacCallum and coworkers [14] found that specific alleles of repeat-containing ORFs prevailed not only in clade 1 (i.e. GPG strains) but also in other groups of genetically similar strains (clades) from mixed sources which they defined using an arbitrary cut-off in MLST-based dendrograms. This would indicate that genetic background may generally have a strong impact on the selection of alleles of repeat-containing ORFs.

We found no evidence that *PNG2* allele variation is used for short-term adaptation to different niches in the host. In this case we would have expected that different alleles predominate in isolates from different body sites. If the same host niche selects different alleles in different genetic backgrounds we may not have been able to see evidence for this selection in the genetically diverse non-GPG strains, but it should have been apparent in the GPG background. Likewise there is no evidence that mutation of *PNG2* is used as a means to evade recognition by the host. In this case the predominant alleles in *C. albicans* populations colonizing a given host would continually change [20], [22] and we would not expect to find alleles predominating in collections of isolates from multiple patients.

In summary *PNG2* appears to be a gene that is capable of generating new alleles at rates comparable to those of contingency genes, but the current data do not support the idea that this mutability is used for short-term (transient) adaptation. The clade-specificity of other repeat-containing *C. albicans* ORFs investigated to date suggests that changes in these proteins are not used in short-term adaptation either. Nevertheless the high number of substantially different protein variants encoded by these repeat-containing *C. albicans* ORFs and the high number of repeat-containing ORFs in the *C. albicans* genome indicate that they play an important role in optimizing the interaction between *C. albicans* and its host in the long-term, with different allele combinations working best in different genetic backgrounds. Only investigations of a higher percentage of all *C. albicans* repeat-containing ORFs will reveal if, and to what degree, the yeast also uses rapid mutation of some of its repeat-containing ORFs for short-term adaptation and evasion of the immune system.

## Materials and Methods

### Strains and Culture Conditions

The strains used in this study are shown in Table 1; they were chosen to represent a collection of 266 infection–causing isolates from 12 geographical regions in 6 countries [23]. *C. albicans* cells were grown at 37°C in YPD medium (2% glucose, 2% Bacto-peptone, 1% yeast extract). For serial transfer experiments, cultures were grown overnight and total cell numbers were determined microscopically using a counting chamber (ZINTL, Western Germany).

### PCR Amplification and Sequencing

All polymerase chain reactions (PCRs) were performed in a final volume of 20 µl containing 1 U *Taq* DNA polymerase (Qiagen Pty Ltd, Clifton Hill Vic, Australia), 4 µl of Q-buffer and 1x PCR buffer supplied by the manufacturer (Qiagen), 10 pmol of each primer, 200 µM of each dNTP (Roche Diagnostics, Auckland, New Zealand), and 10–100 ng DNA. The cycling conditions, varied according to primer sets and the size of the products [46] and included an initial incubation for 2 min at 94°C, followed by 30 cycles of 45 s at 94°C, 45 s at 50–60°C, and 30 s to 3 min at 72°C. All PCR protocols included a final 5 min extension step at 72°C. For colony PCR, a portion of a *C. albicans* colony was picked with a 10 µl pipette tip and mixed with 20 µl PCR reaction mixture; the initial step in the cycling program was altered to 5 min at 96°C. Reactions were carried out in an Eppendorf Mastercycler thermocycler (Eppendorf, Hamburg, Germany). Repeat regions were amplified using primers MC0repf (5′- AACCATCATGACGATCACCA -3′) and MC0repr (5′-GATAAATCTCATCTGCAGGC-3′), and their sizes measured on 5% polyacrylamide gels, using sequenced repeat regions as molecular size standards. The numbers of repeat units were calculated from the sizes of the PCR products. Additional primers used for sequencing were MC0outpf (5′-ACACCGAAGTAGAAGGTGTG-3′), MC0orfpf (5′-ATCTTTTCTTATTTGTTCAAGG-3′), MC0orfpr (5′-ACCCCTTAACTAAGCAATGGC-3′) and MC0sepf: (ACTGAAGCCAAGCCTGCAGA). Locations of all primers are shown in Fig. 1. All nucleotide sequences determined as part of this study have been submitted to GenBank (accession numbers DQ014518 to DQ014537 and GU068595-GU068605).

### Northern Hybridization

For Northern hybridization total RNA was isolated from exponentially growing *C. albicans* cells (1.8×10^9^) by using TRI reagent® (RNA/DNA/protein isolation reagent, Molecular Research Center. Inc., Cincinnati, Ohio, USA) following the manufacturer's instructions. Poly-A mRNA was obtained from total RNA using the Sigma GenElute mRNA isolation kit and the mRNA samples were separated on 1% agarose gels containing 6% formaldehyde, and transferred to nylon membranes (Roche Diagnostics) [46]. The membranes were hybridized with a (^32^P)-labeled DNA probe comprising the repeat region of strain SC5314. The membrane was exposed to an X-ray film as described by the manufacturer (Kodak, Rochester, N.Y., USA).

### Bioinformatics and Statistical Analyses

Kyte/Doolittle hydrophilicity plots and Argos Helix plots predicting transmembrane helices were generated using MacVector 7.2.2 (Accelrys Software, Inc., San Diego, Ca, USA). To determine percent identity among PNGp orthologs, pairwise alignments of amino acid sequences were performed using the BESTFIT and GAP programmes in the Wisconsin Package Version 10.3 (Accelrys Software Inc.). Multiple amino acid sequence alignments were calculated by T-Coffee v2.11 using the web server at http://igs-server.cnrs-mrs.fr/Tcoffee/tcoffee_cgi/index.cgi [47].

To test for purifying selection Paml 3.14 [48] was used initially to calculate the ratio of nonsynonymous to synonymous mutations (dN/dS). Purifying section is indicated by a ratio of nonsynonymous to synonymous mutations (dN/dS) less than one. Paml 3.14 [48] was used to fit two models to the aligned sequence data, one where dN/dS was fixed at 1, and one where it was free to vary. The difference in likelihood between these two models was compared by a likelihood test [40]. The model where dN/dS is free to vary has one extra parameter, so a chi square test with degrees of freedom equal to 1 was used to evaluate if the likelihoods differed significantly between the two models. We did not include the two alleles of SC5314 from the published genome sequence in this analysis. Both of these differed from the SC5314 sequence we determined by the same two point mutations.

A confidence interval for the rate of new allele generation was calculated as the exact (Clopper-Pearson) binominal confidence interval. To calculate how much the frequency of a predominant *PNG2* allele combination should be reduced within 3000 or 100,000 generations, based on the rate at which new alleles were generated in the laboratory, we assumed that in each division 2.8×10^−5^ (or 3.3×10^−6^ or 9. 9×10^−5^) of cells with this combination would undergo a change in one allele, while cells with all other allele combinations could acquire the predominant combination with a probability of 2.8×10^−5^/(number of all observed allele combinations −1). The latter rate is an overestimate because some allele combinations are separated from the predominant allele combination by two changes (when both alleles differ from the alleles that form the predominant combination). However because the rate is low and applies to the minority of the cells without the predominant alleles, this does not significantly affect the results within the timeframe to which the simulation applies. For the same reason, the number of possible allele combinations has little impact on the predicted decline in frequency of the predominant allele combination. Similar calculations were applied to interpret the outcome of serial transfer experiments by Zhao and coworkers [13]. Because some of the repeat regions analyzed by these authors were larger than the *PNG2* repeat region, the rates of new allele generation in the simulation were increased accordingly.
