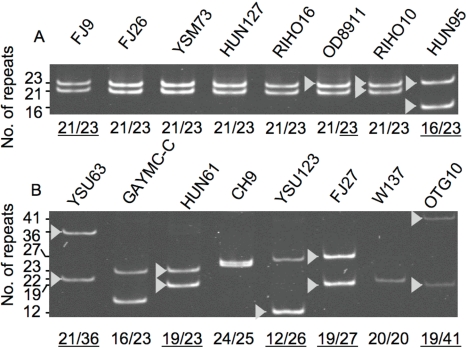# Correction: Impact of Genetic Background on Allele Selection in a Highly Mutable *Candida albicans* Gene, *PNG2*

**DOI:** 10.1371/annotation/a50e86c0-f779-4a4a-80e6-e634a11bbfa8

**Published:** 2010-04-27

**Authors:** Ningxin Zhang, Richard D. Cannon, Barbara R. Holland, Mark L. Patchett, Jan Schmid

Figure 2 was not reproduced correctly due to a problem during generation of a postscript file submitted for production. Please view the correct figure here: 

**Figure:**